# Sensorized Assessment of Dynamic Locomotor Imagery in People with Stroke and Healthy Subjects

**DOI:** 10.3390/s20164545

**Published:** 2020-08-13

**Authors:** Daniela De Bartolo, Valeria Belluscio, Giuseppe Vannozzi, Giovanni Morone, Gabriella Antonucci, Gianluca Giordani, Stefania Santucci, Federica Resta, Franco Marinozzi, Fabiano Bini, Stefano Paolucci, Marco Iosa

**Affiliations:** 1 PhD Program in Behaviour Neuroscience, Sapienza University of Rome, Via Dei Marsi 78, 00185 Rome, Italy; 2 SMART Lab, Santa Lucia Foundation, Via Ardeatina 306, 00179 Rome, Italy; m.iosa@hsantalucia.it; 3 Interuniversity Centre of Bioengineering of the Human Neuromusculoskeletal System, Department of Movement, Human and Health Sciences, University of Rome “Foro Italico”, P.zza Lauro de Bosis 15, 00135 Roma, Italy; v.belluscio@studenti.uniroma4.it (V.B.); giuseppe.vannozzi@uniroma4.it (G.V.); 4 CLEN Lab, Santa Lucia Foundation, Via Ardeatina 306, 00179 Rome, Italy; g.morone@hsantalucia.it (G.M.); g.giordani@hsantalucia.it (G.G.); fede19resta@gmail.com (F.R.); s.paolucci@hsantalucia.it (S.P.); 5 Department of Psychology, Sapienza University of Rome, Via Dei Marsi 78, 00185 Rome, Italy; gabriella.antonucci@uniroma1.it; 6 Department of Mechanical and Aerospace Engineering, Sapienza University of Rome, Via Eudossiana 18, 00184 Rome, Italy; stefania.santucci1990@gmail.com (S.S.); franco.marinozzi@uniroma1.it (F.M.); fabiano.bini@uniroma1.it (F.B.)

**Keywords:** inertial sensors, motor imagery, gait, walking, instrumented movement analysis, neurorehabilitation, stroke

## Abstract

Dynamic motor imagery (dMI) is a motor imagery task associated with movements partially mimicking those mentally represented. As well as conventional motor imagery, dMI has been typically assessed by mental chronometry tasks. In this paper, an instrumented approach was proposed for quantifying the correspondence between upper and lower limb oscillatory movements performed on the spot during the dMI of walking vs. during actual walking. Magneto-inertial measurement units were used to measure limb swinging in three different groups: young adults, older adults and stroke patients. Participants were tested in four experimental conditions: (i) simple limb swinging; (ii) limb swinging while imagining to walk (dMI-task); (iii) mental chronometry task, without any movement (pure MI); (iv) actual level walking at comfortable speed. Limb swinging was characterized in terms of the angular velocity, frequency of oscillations and sinusoidal waveform. The dMI was effective at reproducing upper limb oscillations more similar to those occurring during walking for all the three groups, but some exceptions occurred for lower limbs. This finding could be related to the sensory feedback, stretch reflexes and ground reaction forces occurring for lower limbs and not for upper limbs during walking. In conclusion, the instrumented approach through wearable motion devices adds significant information to the current dMI approach, further supporting their applications in neurorehabilitation for monitoring imagery training protocols in patients with stroke.

## 1. Introduction

Motor imagery (MI) is the mental representation of an action without its physical execution [1] and it represents one of the most promising emergent therapies for motor impairment treatment. This last assumption is based on the functional equivalence theory, supported by many results confirming a functional equivalence between movement and its imagination in terms of time, effort and brain areas involved in both the tasks [2]. Despite its definition and the large amount of studies in which subjects were asked to perform MI decoupled by actions, MI is often accompanied by small movements, especially during kinesthetic imagery (more so than during visual motor imagery), and it may also evoke motor representations involved in balance control [3]. Hence, during MI, subjects partially simulate the imagined movements [4]. Another aspect revealing the intertwinement between MI and sensorimotor systems is the need to adopt a posture congruent with the imagined movement for facilitating the simulation process, also for locomotor imagery [5].

In real practice, such as sports training or rehabilitation, MI does not replace physical exercises and the importance of decoupling MI from the real execution of action decays, allowing the possibility of coupling them to enhance the efficacy of the training [6,7]. For example, a patient with a subacute stroke, who has recovered their standing posture but not yet their walking ability, is often asked by physiotherapist to step in place, move their lower limbs alternatively, and imagine walking, coupling MI and motor actions [6]. This modality of MI accompanied by external movements mimicking in part those mentally represented by the similar temporal and spatial features of the imagined action has recently been proposed and called dynamic motor imagery (dMI) [1,6]. This is conceptually different by the original definition of MI, a condition occurring in the absence of any overt movement. However, the alternative approach of the dMI practice could improve imagination through the movements performed during mentalization, increasing the functional equivalence mentioned above, due to the mimicked movements during the mental practice [7,8].

Recently it has been pointed out that dMI of a walking task may be a suitable tool for recovering locomotor functions in patients with gait disorders of an orthopedic nature [9]. Additionally, neurological patients may benefit from dMI protocols, which are effective in the application of traditional MI in the neurorehabilitation of people with stroke sequelae [10]. Stroke is caused by a total or partial lack of blood supply to the brain, so many different neurological outcomes are possible, including hemiparesis, spasticity, communication disturbances, cognitive deficits or visual–spatial perception disturbances [11,12,13,14]. Among these deficits, bilateral movement coordination is one of the most frequent, generally due to hemiparesis [15]. For the upper limbs, a marked reduction in the use of the affected arm may occur, including the hand, often due to spasticity [16]. For the lower limbs, indeed, hemiparesis and muscle stiffness are often responsible for a loss of balance [17,18,19] and difficulties in walking [20]. Physical rehabilitation is often performed in a framework of a top-down approach which also involves the cognitive aspects of movements, especially for patients in the subacute phase of stroke [10]. It deals with functional equivalence principle-based rehabilitation protocols, such as mirror therapy, actions observation therapy, and motor imagery, also supported by using new technologies [21].

Despite few results reporting that stroke patients may have some deficits in terms of motor imagery [6], it has been suggested that MI protocols can enhance the rehabilitation outcomes, and in turn can be improved by rehabilitation itself in a mutual circular process [22]. In this scenario, it could be important to measure the motor imagery ability, and many different protocols were suggested for measuring motor imagery performances, such as hand laterality tests or mental chronometry, but most of them were based on a single body part, mainly the hand [23]. Some mental chronometry tests were also tested in patients with stroke during static and dynamic motor imagery related to locomotion [6].

The use of dMI exercises may also allow the assessment of MI performance, not only from a temporal point of view, but also considering its spatial features. In fact, considering all the above considerations, it is of great importance to objectively verify whether people experiencing a stroke are also able to imagine complex coordinated actions such as the limb oscillations during locomotion. Limb oscillations could be modeled as those of a pendulum, in which the optimal frequency of oscillations depends on the limb length, but not on its weight, according to the physical law of isochronism [24]. Applying this law, that frequency is expected to be proportional to the root square of the ratio between the acceleration of gravity and leg length. However, during walking, all the four limbs oscillate at the same frequency, with the movements of body that could be modeled as an inverse pendulum [24]. Previous findings showed that these frequencies are lower than 1 Hz (0.85 Hz for the leg, 0.98 for the upper limb), not so far from the stride frequency of human gait (0.83 Hz at 1.3 m/s) [25].

Many studies have already investigated coordinated movements in stroke patients, often focusing alternately on the upper limbs (for example bimanual tasks [26]) or on the lower limbs (locomotion [27]), or on the alteration of the above-described pendular mechanism [24]. Few studies have so far dealt with the coordination of all four limbs while walking in stroke survivors and a healthy comparison sample [28,29,30]. A simple action such as walking implies a complex coordination: arms are moved out-of-phase with each other at a frequency that is synchronized with the stride frequency and in-phase with the contralateral lower limb [31]. Studies on stroke patients have focused on the degree of synchronization of the affected arm during locomotion, especially using cognitive stimulation as acoustic feedback [32,33]. Despite the emerging literature about the use of MI and dMI, all the assessment protocols were mainly related to the mental chronometry of single gestures, and hence focused on temporal performance. Semi-quantitative clinical scales are also used in the dMI assessment, with the main limitation of not allowing to verify directly whether and how the patient is imagining the required movement. In the literature, there is a lack of quantitative assessments on locomotor imagery related to limb oscillations. In fact, many features should be measured for assessing the capacity of imagery of complex coordinated actions, such as locomotion. Parameters such as the angular speed, frequency and waveform of limb movements could be easily quantified during dynamic motor imagery protocols using wearable inertial sensors [34].

With respect to MI, the assessment of imagery performance during dMI could be easier, because the accompanied movement could be quantitatively assessed using wearable inertial devices [34,35].

The aim of the present study is to investigate the capacity of patients with stroke to imagine walking, mentally reproducing the actual movements they do during real walking, in particular the limb oscillations. For this purpose, the dMI allowed to measure, by wearable inertial sensors, the limb oscillations performed by patients mimicking in place those usually performed during walking. The comparisons of upper and lower limb oscillations during dMI with those performed in simple motor tasks without any imagery and with those performed during actual walking could allow to test motor imagery in patients. Their data were compared with those of age-matched healthy adults as controls and those of young adults as a physiological reference. Our hypothesis is that single limb swinging is poorly related to the swinging of the same limb during walking, when coordinated among all the four limbs, but this difference can be reduced by dMI, in terms of waveform, angular speed amplitude and frequency of movements.

## 2. Materials and Method

All measurements and experimental conditions have been performed in accordance with the Declaration of Helsinki standards. The study was approved by the Local Independent Ethics Committee of IRCCS Santa Lucia Foundation (Rome, Italy) (protocol number: CE/PROG.738). Every subject provided written informed consent prior to participation. We recruited both healthy subjects and patients with stroke at the Private Neurorehabilitation Unit of Santa Lucia Foundation.

### 2.1. Participants

To investigate the physiological performances, the possible age-related decline, and pathological performances affected by stroke, three groups were enrolled, as in a previous study on locomotor imagery [6]: a first group of 27 young healthy participants (YG); a second control group consisting of 15 healthy older adult participants (CG), and, finally, a group of 15 patients with cerebral stroke admitted to our hospital for neurorehabilitation (PG). The sample size for each group was determined on the basis of data from a previous study [6] on a sensorized assessment of dMI in which 12 subjects were enrolled in each one of the two healthy groups and 20 in the stroke group, increasing these samples in our study by at least 25% to improve the power of the analyses. We collected demographical, clinical, and anthropometric data for all the enrolled subjects. For the PG group, the inclusion criteria were: (1) being able to stand independently (no aid or physical support) for at least 30 s; (2) to climb a step being able to lean on a support but without help from the physiotherapist; (3) being able to walk independently on a 10 m walkway without help; (4) not report any cognitive impairment or linguistic difficulties, in order to understand and follow the instructions for the experiment. For the PG group, only patients who had experienced a stroke event at least three months before the data collection were included in the study.

For the YG and CG subjects, the inclusion criteria were: (1) be of legal age; (2) have Italian as their mother tongue or be resident in Italy for more than ten years; (3) not have sensory or motor deficits or orthopedic problems that can influence motor performance.

### 2.2. Procedures

#### 2.2.1. Clinical Testing

Patients were clinically evaluated through tests and scales commonly used for assessing the most frequent stroke deficits [36]: gait ability was assessed by the functional ambulation category (FAC) and Tinetti scale (TS-Walk), static balance was assessed by the Berg balance scale (BBS), the degree of independence in the activities of daily living was assessed by the Barthel Index (BI), the upper and lower limbs motility was assessed by the Motricity Index (MI) and hand dexterity was assessed by a Box and Blocks test (requiring transport from a box to another one over a partition, as quickly as possible, with the largest number of 2.5 cm^3^ wooden blocks [37]). Participants were also tested on their ability to imagine movements through the Kinaesthetic and Visual Imagery Questionnaire (KVIQ), a questionnaire used to determine the extent to which individuals were able to visualize and feel imagined movements. The KVIQ assesses, on a five-point ordinal scale, the intensity of the sensations (kinesthetic: K subscale) and the clarity of the image (visual: V subscale) that the subjects are able to imagine.

#### 2.2.2. Motor Tasks and Setting

**Figure 1:**
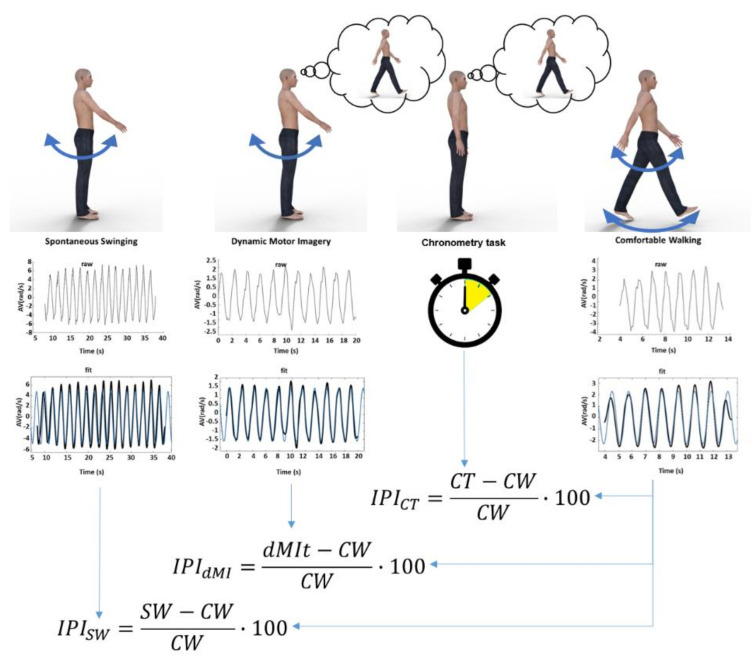
Schematic representation of the study**.** At the top of the figure, the four experimental conditions are represented: limb swinging (SW), limb swinging during dynamic motor imagery (dMI), chronometry task of imagined walking without any movement (CT) and comfortable walking (CW). In the middle: plots showing a raw signal of the angular velocity recorded, plots showing filtered signals (black lines) and sinusoidal fits (blue lines) for SW, dynamic motor imagery task (dMIt), CW, whereas just time was measured with a stopwatch in CT. Below, the formulas of the Incongruency Performance Index for the first three experimental conditions compared to the actual walking performance of the fourth condition.

The experimental procedure is made up of four conditions (as shown in Figure 1): (1) single limb swinging without any cognitive task (SW); (2) single limb swinging during imagining walking (dynamic motor imagery task, dMIt); (3) a mental walking chronometry task without any actual movement (CT); (4) comfortable actual walking (CW). The four conditions were performed four times, one for each limb. The sequence of limbs was randomized among subjects (for avoiding a transfer effect from one limb to another; to avoid that swinging without imagery could be affected by imagery and imagery by actual movements), in accordance with the protocols used in previous studies [6,38,39].

Before starting the experiment, instructions on how to perform the experimental tasks were provided. However, in order not to affect their performance, none of the participants had been explicitly told about the purpose of the study, namely that we wanted to investigate the limb oscillations.

An experimental setting was created for carrying out the motor tasks. Condition SW was the first one recorded. Each participant was asked to be in a standing posture on a step stool (height 10 cm) and to oscillate one limb in the sagittal plane in each trial. Four trials were performed (one for each limb) in a randomized order given by the researcher, and each trial lasted 30 s. During the oscillations of a lower limb, the subject was the on the step with their contralateral leg, whereas the oscillating leg is moved in the sagittal plane in the air. To avoid losing balance, participants required handles to lean on which were on a braked walker positioned in front of them with handles close to the subject’s hands.

Subsequently, the dMIt was recorded. The tasks were the same of SW, but now subjects were also asked to imagine walking during limb swinging (the request was “oscillate this limb imagining as you are doing it during walking”). No indication was given regarding maintaining open or closed eyes. In the CT condition, we asked the participants to position themselves in front of the first target (start) and to imagine with open eyes the time necessary to reach the second target (stop) placed on the straight path, without performing any movements. No information was given about the length of the path. The examiner synchronized the start of the stopwatch with a vocal start at which the participant began to imagine, the stopwatch was stopped when the participant verbally stated that he/she had mentally reached the second target [6,38]. The last task (CW) consisted of the actual recording of the movement of the four limbs during a comfortable walk along the 10 m-pathway, marked by two pins used as start and stop targets. The duration of this performance was compared with that estimated by the subjects in the CT task (assessed by using a Stopwatch App for iOS [6]), thus being able to compare the subjects ability to temporally estimate the movement with their actual performance.

### 2.3. Apparatus of Wearable Inertial Sensors and Inertial Data Processing

For recording the movements, each participant was equipped with four synchronized magneto-inertial measurement units (MIMUs) (128 Hz, Opal, APDM, Portland, OR, USA), placed on both ankles and wrists. The two MIMUs placed on the right and left wrists were positioned between the ulna bones and the radius, while those on the right and left ankles were positioned slightly above the lateral malleoli. The MIMUs were securely fixed to participants’ bodies using multiple elastic velcro straps that adjust to fit different body sizes. These fixing devices allowed the prevention of any possible device displacement from its initial location. The MIMUs were located on the anatomical district and then carefully aligned to better approximate the cranio-caudal, medio-lateral and antero-posterior axes of the body segments. To guarantee a repeatable system of reference for all participants, an extra sensor was located on the floor, with one axis aligned with the direction of progression and another axis parallel to the gravity vector. A 5 s trial was recorded at the beginning of each trial when the participant was asked to be firm maintaining standing position. This static acquisition allows for the computation of the orientation of the current local frame and then to rotate the local reference systems via software through a rigid transformation on the basis of the extra-sensor orientation, in order to obtain an anatomical reference frame defined by the sagittal plane and its orthogonal axis [40]. Each MIMU embedded three-axial accelerometers and gyroscopes (±6 g and ±1500°/s of full-range scales, respectively) and provided the quantities with respect to a unit-embedded system of reference. The raw data recorded by the MIMUs were transmitted via Bluetooth and stored in a computer.

The raw data processing was performed using the MATLAB software (The MathWorks Inc., Natick, MA, USA). The analyzed data referred to those obtained by gyroscopes along the sagittal plane. Data were pre-processed and digitally low-pass filtered, using a fourth order Butterworth filter with a cut-off frequency of 2 Hz. This cut-off frequency was chosen according to the Nyquist theorem, and based on above reported data related to limb oscillations and stride frequencies that are lower than 1 Hz in the human gait [25]. As the required oscillations were mainly sinusoidal, such as those of a pendulum, according to the swinging tasks asked to the subjects, to obtain the speed amplitude and frequency of movements, the filtered angular velocity data were fitted with a sinusoidal curve y=αsin(bx+c) using a least square method (we preliminarily verified that the results of this approach were not significantly different from those obtained from the first harmonic of a fast Fourier transformation, *p* > 0.6). The curve obtained from the sinusoidal fit was analyzed, allowing for the characterization of swinging movements by the following three parameters: AV = *a*, the amplitude of the angular velocity of movement (rad/s); Fq = *b_1_*/(2π), the movement frequency (Hz); the similarity to a sinusoidal waveform computed by using the coefficient of determination R-squared (R^2^) of the sinusoidal fit.

### 2.4. Indices of Motor Imagery Performance

According to the literature on the Imagery Performance Index (IPI) [35], the similarity of movement parameters was assessed comparing the parameters measured during dMI with respect to those of actual movements. The IPI is calculated as the relative difference between the performance during mental imagery and the relevant parameters of the performance recorded during the actual execution of the locomotor task, expressed as a percentage of the actual execution. In the same way, we computed the IPI for the sinusoidal fit parameters (amplitude of movement velocity, frequency of movement and waveform similarity obtained from the R-squared values). We computed the IPI also comparing limb oscillations during the SW and CW condition, for comparing with that computed between dMIt and CW. So, because of computed also for a task not including imagination (SW), and because higher percentage values correspond to a higher incongruity with respect to the actual walking, we refer to the IPI as an Incongruity Performance Index. Figure 1 shows the protocol of the study in an exemplificative manner for the four conditions (SW, dMIt, CT, CW) and the successive data analysis, and finally the computation of Similarity Performance Indices.

### 2.5. Statistical Analysis

The statistical analyses were performed using the version 23 of IBM SPSS statistical software. Data were summarized in terms of the median and interquartile range (third less first quartile) for the clinical scales, mean and standard deviation for continuous measured parameters, and the mean and standard error of the mean for the IPI percentages. The normal distribution of each parameter was verified using the Shapiro-Wilk test. For normally distributed data, an analysis of variance was performed followed by *t*-tests as post-hoc comparisons. For data that was not normally distributed (R^2^ and IPIs), nonparametric statistics were used for inferential analyses. A Krukal-Wallis analysis was used to perform between group comparisons among the data of YG, CG and those of PG divided by the affected an unaffected side (in relationship to hemiparesis), followed by post-hoc analyses performed with Mann-Whitney u-tests corrected for Bonferroni. A Wilcoxon test was used to compare SW and dMIt IPIs within each group. The Spearman coefficient was used to assess the entity of possible correlations between the frequency of leg or arm oscillations and the root square of the ratio between the acceleration of gravity and leg or arm length. For all the tests, the significance level was set at 0.05, with the exception of post-hoc analyses (alpha = 0.0167) and correlations, for which the alpha level of significance was divided by the number of possible comparisons (12) and set at 0.00417, according to Bonferroni correction.

## 3. Results

As shown in Table 1, no statistically significant differences were found between the PG and CG in terms of demographical (age: *p* = 0.096 *t*-test, gender: *p* = 0, chi squared test) and anthropometric (*p* > 0.05) characteristics. The clinical assessment of patients, which depicts the level of their deficits, is reported in Table 2. For 80% of patients, it was a first ictal event and in 53% of cases it was an ischemic stroke. With respect to the dMIt tasks, only four subjects (two of them belonging to the CG group, the remaining to the PG group) decided to perform these trials with closed eyes. A clinical assessment was performed only for PG, and the only scale administered also to healthy subjects was the Kinesthetic and Visual Imagery Questionnaire: the median (and interquartile range (IQR)) value for YG was 70 (26) and for CG was 82 (31).

**Figure 2:**
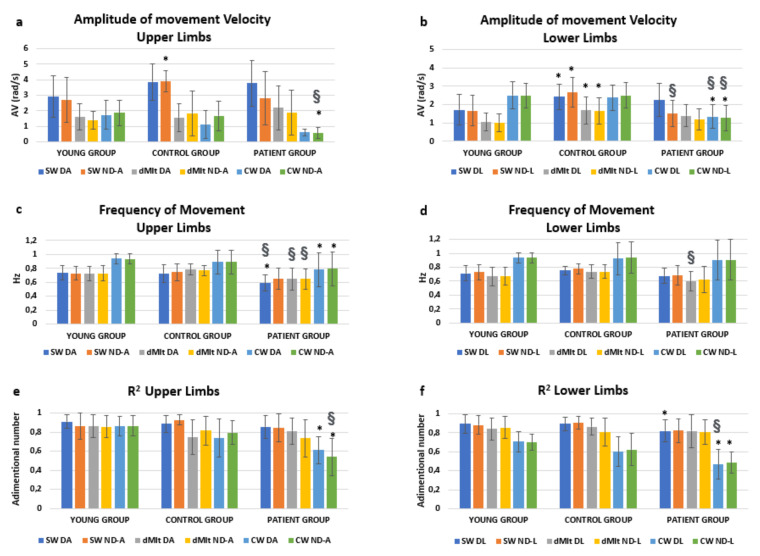
Means and standard deviations of the computed parameters (amplitude of movement velocity, computed for upper (**a**) and lower (**b**) limbs; frequency of movement, upper (**c**) and lower (**d**) limbs; R^2^, upper (**e**) and lower (**f**) limbs), obtained analyzing the angular velocity measured by gyroscopes embedded in magneto-inertial measurement units (MIMUs). Experimental conditions are: SW (swing oscillation), dMIt (dynamic motor imagery task), CW (comfortable actual walking), while upper and lower limbs are indicated as D (dominant or unaffected arm, respectively in healthy and pathological subjects), ND (nondominant or affected arm) A (arm) or L (leg). Post-hoc statistically significance differences were reported with respect to the YG (*) or between the PG and CG (§).

Performance data are reported in Figure 2 with the relevant results of post-hoc analyses. We were mainly interested in the effects of condition * group. This interaction resulted significantly affecting the amplitude of the oscillation speed (F = 14.534, *p* < 0.001), but not its frequency (F = 1.107, *p* = 0.357). Interestingly, a significant interaction was found also for condition * group * limb (F = 2.521, *p* = 0.045) for the speed amplitude. In fact, as shown in Figure 2, the amplitude of speed was reduced in dMIt with respect to SW, becoming more similar to CW for upper limbs. The same occurred in patients for lower limbs, but the reduction in speed during dMIt did not correspond to the values of actual locomotion for healthy subjects.

As shown in Figure 2, oscillations were characterized by higher frequencies during actual walking than in all the other conditions, for all the three groups. To test if these frequencies could be related to the pendular mechanism for upper and lower limbs, the correlations between limb length and the oscillation frequencies were tested.

In the YG and CG, the mean frequency of lower limb oscillations was found to be significantly correlated with the root mean square of ratio between gravity acceleration and leg length in CW (R = 0.318, *p* = 0.003) and SW (R = 0.315, *p* = 0.004), but not in dMIt (R = 0.286, *p* = 0.008, not significant with the Bonferroni correction). Interestingly, in healthy subjects, the frequency of upper limb oscillations was found to be significantly correlated with the root square of ratio between gravity acceleration and arm length in CW (R = 0.321, *p* = 0.003) and dMIt (R = 0.360, *p* = 0.001), but not in SW (R = 0.290, *p* = 0.007). In CW, the frequency of upper limb oscillations was found significantly correlated with that of lower limbs (R = 0.862, *p* < 0.001). Conversely, for patients during actual walking, neither the frequency of oscillations for upper limbs (R = −0.273, *p* = 0.159) or for lower limbs (R = 0.230, *p* = 0.240) were significantly correlated to relevant anthropometric measures.

## 4. Discussion

The main aim of the study was to measure objectively the ability to imagine a locomotor task in patients who have experienced a stroke with respect to healthy subjects, using inertial sensors. The interest was in evaluating how much the limb oscillatory speed amplitude, frequency and waveform were similar when performed in place without and with a locomotor imagery task to the same variables measured during actual walking. Considering the scientific data previously reported on neurorehabilitation [6,38,39], a further purpose was to establish whether or not stroke patients are capable of imagining locomotion in a dynamic way and how much their imagination corresponds to real performance. Given the need to compare imagery with a real walking performance, the patients enrolled in our study were not severely affected, as shown in Table 2. It could be at the basis of the fact that the IPI reported in the last row of Table 3, when CT was compared to CW, was not significantly different between healthy subjects and patients. For the three groups, these IPIs ranged between –2% and 3%, with a *p*-value of 0.941, showing similar performances among subjects. This result is in line with previous studies, showing that, for forward comfortable walking, the performance of patients less severely affected by stroke during a dynamic motor imagery task can reproduce well, in terms of time, their actual walking performance [6]. However, the innovative aspect of our study was the movement analysis of single functions, id est the limb oscillations, not only the global temporal performance.

As shown in Table 3, our hypothesis that dMI could help to increase the similarity with actual walking oscillations was confirmed by most of the parameters and groups. In fact, we found the dMI reduced the IPI percentages of speed amplitude for the upper limbs of all the three groups and for the lower limbs of patients (both affected and unaffected limbs), for the frequency of unaffected upper limbs of patients, for the waveform in the upper and lower limbs of young and older adult subjects (CG) and affected upper limbs of patients. However, in some cases, during the dMI-task the IPI significantly increased for lower limbs: in terms of speed amplitude for young and older adult subjects, and in terms of frequency for young subjects and patients (both the affected and unaffected side). These results could be summarized by reporting that dynamic motor imagery seemed to be effective at reproducing the actual walking movements of upper limbs more than those of lower limbs. This result seems to be not dependent on age, despite the higher IPI values found in healthy older adults than in young subjects. Age-related performance differences in motor imagery were already observed in functional magnetic resonance imaging studies [41]. Young subjects showed a more automatic motor behaviour [41], with a mutual inhibitory sensory interaction [42], whereas older adults showed a functional activation of the supraspinal locomotor networks, ensuring a greater multisensory cortical activation, potentially related to a lacking compensation of efficiency, but also showing compatibility with reduced reciprocal inhibitory sensory interactions during locomotion [41].

According to the above findings [41], a possible explanation for the differences between upper and lower limbs found in our study is related to the fact that lower limb movements during walking are strictly linked to automatic inverted pendulum oscillations (as shown by the significant correlation between the frequency of lower limb oscillations and the root mean square of ratio between gravity acceleration and leg length in CW and SW, but not in dMIt). This automaticity might reduce the access of motor imagination for mimicking these oscillations. In fact, it has been shown that healthy subjects could have difficulties in reproducing harmonic behaviors usually implemented unconsciously [43]. Gait is characterized by a rhythmic behavior with phases and sub-phases alternated at a certain frequency [44,45,46] and depends on the activations produced by autonomous central pattern generators (CPGs) probably located in the spinal cord [47,48]. Despite some studies reporting that CPGs guarantee the necessary muscle activation patterns independently from peripheral feedback [49], other results supported the importance of sensory feedback and stretch reflexes in walking models [50].

Furthermore, during walking, the effects of the ground reaction force could be difficult to be explicitly imagined and reproduced in dMIt. People reproducing walking movements in air showed significantly different foot trajectories than when walking was performed on the ground (even if the ground reaction force was reduced by 95%) [51]. The importance of the surrounding environment with which the subject may interact in an actual or imagined condition was already reported by previous studies on motor imagery and how motor imagery may affect distance perception [38,52]. Conversely, dMI could be easier for upper limb movements, for which the only external force is the gravitational one. The improvement in the lower limb speed amplitude IPI for patients could indirectly confirm the suggested hypothesis, because for them the fluid pendular mechanism of walking is lost. Another result supporting this hypothesis is the correlation between the leg length and lower limb oscillation frequency. The pendular mechanism of walking allows healthy subjects to recover energy during locomotion [24]. For the law of isochronism of pendulum, the theoretical oscillation frequency only depends on the length of limbs, not on their mass. A surgical or artificial change in leg length altered the spatio-temporal gait parameters [45], as well as the locomotor body schema [53]. It supports the idea that the motor imagery can be strictly intertwined with the locomotor body schema [53].

In patients, the deficits due to stroke may have greatly impaired the movements and reduced the capacity of exploiting the pendular mechanism of walking [54]. It may explain our results. According to the functional equivalence theory, a more difficult task has been imagined as it requires more time, more effort, and is executed slower [2]. However, patients with stroke showed that they may overestimate their motor abilities during static motor imagery, probably for some bias related to the motor schemas tailored before stroke, but this bias is reduced in dynamic motor imagery tasks when movements are mimicked during motor mentalization [6]. The time elapsed from the stroke and the rehabilitation practice may allow patients to “update” their locomotor schema in which information on the motor deficit has flowed. This is also in line with the results of a previous study about the locomotor body scheme [53] which compared the performance of a subject before and immediately after undergoing a limb length surgical elongation. It is plausible that patients became progressively more aware of their motor impairment because it required them to pay more attention and plan the movements necessary for the execution of complex motor behaviors, such as locomotion. On the other hand, when cognitive resources are engaged in the constant planning of movement, this in turn can affect the harmony and fluidity of movements that appear more rigid [55].

Considering the overall results about healthy subjects and patients, at this point, an instrumented movement analysis during dynamic motor imagery seems to be important for objectively measuring the ability of patients to imagine the realization of the motor gesture and to compare it with the actual locomotor execution. The sensory feedback should be included in motor imagery training protocols, for improving the equivalence of dMI with actual gestures. In the future, rehabilitative programs could provide the application of biofeedback during motor imagery in order to improve patient’s performance during locomotion, especially for lower limbs. For upper limbs, this approach was already suggested using a brain–computer interface during motor imagery [56], whereas more simple wearable devices during dMI could be used for locomotion, a condition in which the use of brain-computer interface is limited.

The definition of MI, id est the mental representation of an action without its physical execution [1], seems to derive from the protocols used to test the functional equivalence between MI and motor execution. However, in real life, MI is often accompanied by some actual movements, such as when a sportsman imagines performing a gesture and mimics it on the spot in the air, or when a patients confined to a wheelchair tries to move their lower limbs when they imagine walking. Many experimental findings have put back together these two actions, the mental one and the motor one. In fact, MI may depend on the posture of the subject, being more effective when the subject is in a posture congruent with the task to imagine [5], and, in turn, affect the posture, causing possible balance alterations and small movements [3]. In our study, all subjects were stood upright, congruent with the walking task, and they could oscillate their limbs, also their lower ones, in the air. However, the results of our study should be read despite its limits. For example, though we recorded how many subjects performed the SW and dMIt with closed eyes, which was a small group, the effects of vision or balance stabilization was not taking into account in this study, but further investigations may involve these aspects. Furthermore, as stated above, the limbs, including the lower ones, were swung in the air on the spot, without the physical interaction with the ground, and it may be at the basis of the difference between MI and real execution in upper vs. lower limbs. Further studies are needed to investigate this aspect deeper, as well as the role of posture, balance stabilization and vision.

## 5. Conclusions

Given the purpose of this study, MIMUs were fruitfully used for assessing the motor behaviours of healthy and stroke subjects. Previous studies used a single MIMU for comparing dMI and actual walking [6,39], but in our study the use of more MIMUs allowed for the above comparison of the single limb swinging analysis. The instrumented movement analysis approach allowed to objectively quantify the single limb movements during the dynamic motor imagery task and to compare this performance with those performed during the actual walking in order to estimate the participants’ ability to imagine single movements involved in human locomotion. This approach allowed to identify statistically significant differences regarding the effects of dMI on limb oscillations, despite the absence of any significant differences in the chronometric task.

So, this method of investigation allowed an objective and sensitive evaluation of how patients imagine their movements during mental representation of walking. Upper limbs seemed benefit more from dMI than lower ones, probably because the latter were moved in a more automatic way that also depends on sensory feedback, reflexes and ground reaction force. The importance of upper limbs oscillations was already reported in the scientific literature for a correct implementation of locomotor schemes [57].

Taking up the concept of the famous neuropsychologist Luria about kinetic melodies [58], the movement can be considered a consequential harmonic structure in which each single motor command has been embedded, and each movement must be performed with the right timing, similarly to each instrument of an orchestra. The identification of spontaneous frequencies in single limb movements and their stimulation, through cognitive techniques, could represent an effective training method to restore the harmony of movement in stroke patients.

In conclusion, our findings showed that dynamic motor imagery may positively influence locomotor movements both in healthy and pathologic people, more in upper than lower limbs. However, this study did not deal with a treatment based on dMI. Further studies should investigate whether the use of dMI protocols involving coordinated movements based on paired (in-phase and out-of-phase) oscillatory movements of multiple limbs together can help restore harmonious movements in stroke patients. In the light of our results, rehabilitation protocols based on dMI should include the contact with the ground when lower limb movements are mimicked and the measure of limb movements by wearable devices for monitoring the congruence of patients’ imaginations with the actual execution of movements.

## Figures and Tables

**Table 1:** Demographic and anthropometric characteristics of the young group (YG), the control group (CG), and patient group (PG). Measures are summarized as the mean ± standard deviation values. Last columns report the *p*-values for the comparison of CG and PG.

	YG	CG	PG	*p*-Value
Nr. of Participant	27	15	15	-
Nr. of Males	13	8	9	0.715
Age (years)	25.1 ± 3.6	54.2 ± 15.3	53.9 ± 17.1	0.964
Stature (cm)	171.2 ± 7.8	168.3 ± 7.7	174.1 ± 8.8	0.065
Upper Limb Length (cm)	58.3 ± 3.5	57.3 ± 3.2	59.7 ± 4.2	0.723
Arm Length (cm)	32.2 ± 2.6	30.3 ± 2	31.4 ± 2.2	0.175
Forearm Length (cm)	26.6 ± 1.7	26.9 ± 1.4	28.3 ± 2.3	0.059
Lower Limb Length (cm)	81.15 ± 3.9	81.3 ± 5.1	85.14 ± 6.6	0.089
Thigh Length (cm)	40.4 ± 4.8	43.6 ± 3.4	46.1 ± 4.5	0.105
Shank Length (cm)	40.7 ± 3	37.6 ± 3.7	39.07 ± 3.1	0.281

**Table 2:** Median and interquartile range (IQR) for the scores obtained at clinical scales and tests.

Clinical Assessment of Patients	Median	IQR
Times from stroke Functional Ambulation Categories	37 4	70 1
Tinetti Scale-Walk	11	2
Berg Balance Scale	54	3.5
Barthel Index	100	2.5
Motricity Index—Affected Upper Limb Motricity Index—Not Affected Upper Limb Motricity Index—Affected Lower Limb Motricity Index—Not Affected Lower Limb	83 99 83 99	18.5 0 13 0
Box and Blocs—Affected Hand Box and Blocs—Not Affected Hand	30 47	34 11.5
Kinesthetic and Visual Imagery Questionnaire	97	22

**Table 3:** Incongruity Performance Index (IPI) in the young group (YG), control group (CG) and patients for the unaffected and affected side (PG), for the upper and lower limbs, that compares to actual locomotion during the swing task (SW) and the dynamic motor imagery task (SW and dMIt, respectively). The row dMIt vs. SW report the *p*-values of the Wilcoxon test within subjects for each group, the last row reports the IPI between the time spent during the mental chronometry task (CT) and comfortable walking (CW), the last column reports the between-groups comparison performed by a Kruskal–Wallis analysis. In bold are the statistically significant *p*-values.

Parameter	Limbs	IPI		YG	CG	PG Unaffected	PG Affected	Between-Groups *p*:
Speed Amplitude	Upper Limbs	SW		−111 ± 26%	−340 ± 76%	−578 ± 98%	−494 ± 91%	<**0.001**
		dMIt		−16 ± 18%	−68 ± 24%	−298 ± 80%	−278 ± 59%	<**0.001**
		dMIt vs. SW	***p***	<**0.001**	<**0.001**	**0.002**	**0.006**	-
	Lower Limbs	SW		13 ± 12%	−9 ± 6%	−169 ± 75%	−28 ± 12%	<**0.001**
		dMIt		52 ± 6%	30 ± 6%	−42 ± 29%	−8 ± 12%	<**0.001**
		dMIt vs. SW	***p***	**<0.001**	**<0.001**	**0.001**	**0.012**	-
Frequency	Upper Limbs	SW		22 ± 1%	16 ± 3%	18 ± 8%	9 ± 10%	0.238
		dMIt		22 ± 1%	11 ± 2%	9 ± 9%	11 ± 9%	**0.001**
		dMIt vs. SW	*p*	0.901	0.056	**0.020**	0.910	-
	Lower Limbs	SW		23 ± 1%	14 ± 3%	20 ± 6%	20 ± 6%	0.112
		dMIt		28 ± 1%	18 ± 3%	29 ± 7%	27 ± 8%	**0.014**
		dMIt vs. SW	***p***	**0.002**	0.120	**0.001**	**0.027**	-
Waveform	Upper Limbs	SW		−4 ± 2%	−30 ± 12%	−49 ± 12%	−95 ± 36%	<**0.001**
		dMIt		−1 ± 3%	−10 ± 9%	−42 ± 12%	−56 ± 20%	<**0.001**
		dMIt vs. SW	***p***	**0.012**	<**0.001**	0.112	**0.017**	-
	Lower Limbs	SW		−29 ± 3%	−55 ± 7%	−97 ± 23%	−79 ± 14%	<**0.001**
		dMIt		−22 ± 3%	−44 ± 7%	−97 ± 24%	−76 ± 13%	<**0.001**
		dMIt vs. SW	***p***	**0.014**	**0.028**	0.776	0.910	-
Chronometry Task		dMIt		3 ± 5%	−2 ± 9%	2 ± 16%		0.941
